# HIF-1α-dependent upregulation of angiogenic factors by mechanical stimulation in retinal pigment epithelial cells

**DOI:** 10.1242/dmm.050640

**Published:** 2024-05-01

**Authors:** Atsushige Ashimori, Fumiaki Higashijima, Tadahiko Ogata, Ayano Sakuma, Waka Hamada, Junki Sunada, Ren Aoki, Masanori Mikuni, Ken'ichiro Hayashi, Makiko Wakuta, Takuya Yoshimoto, Akira Minamoto, Ji-Ae Ko, Kazuhiro Kimura

**Affiliations:** ^1^ Department of Ophthalmology, Yamaguchi University Graduate School of Medicine, 1-1-1 Minami-Kogushi, Ube City, Yamaguchi 755-8505, Japan; ^2^ Department of Ophthalmology and Visual Science, Graduate School of Biomedical and Health Sciences, Hiroshima University, 1-2-3 Kasumi, Minami-ku, Hiroshima 734-8551, Japan

**Keywords:** Age-related macular degeneration, Angiogenesis, HIF-1α, Mechanotransduction, Retinal pigment epithelial cells

## Abstract

Mechanical stimulation as a mimic of drusen formation in the eye increases the expression of angiogenic factors in retinal pigment epithelial (RPE) cells, but the underlying molecular mechanisms remain unclear. We investigated and characterized the effects of mechanical stimulation on the expression of angiogenic factors in RPE cells both *in vitro* and in a mouse model. Mechanical stimulation increased the expression of vascular endothelial growth factor (VEGF, encoded by *VEGFA*) and other angiogenesis-related genes in cultured RPE1 cells. The presence of hypoxia-inducible factor 1α (HIF-1α, encoded by *HIF1A*) was also increased, and both knockdown of HIF-1α and treatment with the HIF-1α inhibitor CAY10585 attenuated the effect of mechanical stimulation on angiogenesis factor gene expression. Signaling by the tyrosine kinase SRC and p38 mitogen-activated protein kinase was involved in HIF-1α activation and consequent angiogenesis-related gene expression induced by mechanical stimulation. Our results suggest that SRC–p38 and HIF-1α signaling are involved in the upregulation of angiogenic factors in RPE cells by mechanical stimulation. Such *in vivo* suppression of upregulated expression of angiogenesis-related genes by pharmacological inhibitors of HIF-1α suggests a new potential approach to the treatment of age-related macular degeneration.

## Teaser

**Editor's choice:** Mechanical stimulation of retinal pigment epithelial cells *in vitro* and in a mouse model increased the expression of *VEGFA* and other angiogenesis-related genes in a HIF-1α-dependent manner.

## INTRODUCTION

Retinal pigment epithelial (RPE) cells play an important role in maintaining retinal homeostasis (Lakkaraju et al., 2020). Located between the retinal photoreceptor cells and the choroid, RPE cells perform a variety of essential functions including transport of nutrients, removal of waste products and absorbance of excess light (Blasiak et al., 2019; Boulton and Dayhaw-Barker, 2001; Chichili et al., 2005; Seagle et al., 2006; Strauss, 2005). In addition, RPE cells act as a barrier between the blood circulation of the choroid and that of the neural retina, forming the outer blood–retinal barrier (Campbell and Humphries, 2012; O'Leary and Campbell, 2023). The retinal pigment epithelium is also subjected to various stresses, both external and internal. External stresses include blue-light hazard, oxidative stress, toxic compounds (e.g. hydroxychloroquine, aminoglycosides, etc.) and inflammation, whereas internal stresses result from gene mutations or age-related changes that affect RPE cell function. These factors can give rise to retinal pigment epithelium dysfunction and cellular damage, leading to the development and progression of retinal disease (Chen et al., 2009; Gu et al., 2012). Among such diseases, age-related macular degeneration (AMD) is one of the most common and serious. In AMD, the accumulation of waste products in the retinal pigment epithelium, drusen and pachychoroid contribute to retinal pigment epithelium dysfunction, abnormal angiogenesis and photoreceptor cell death, potentially leading to severe impairment of vision (Chen et al., 2016; Somasundaran et al., 2020). Some AMD conditions form drusen and others do not, but the causes of the differences between the two have not been clarified. AMD that forms drusen involves mechanical environmental changes to the RPE cells due to pressure associated with lipofuscin accumulation and scaffold instability.

Mechanical stimulation plays a role in many biological processes such as cell differentiation and metabolism, and it has been implicated in various diseases including cancer and inflammatory conditions (Bissell et al., 1982; Ingber et al., 2014; 1981; Wang, 2017). Cells sense mechanical stimulation through mechanosensors that include the cytoskeleton, ion channels and Piezo1 (Elosegui-Artola et al., 2017; Ge et al., 2015; McMurray et al., 2015; Tamada et al., 2004). These sensors then trigger various conventional signaling pathways in a manner thought to depend on factors such as cell type, morphology and scaffold stiffness (Paradigm et al., 1993; Takahashi et al., 2009; Weinbaum et al., 2011).

The expression of vascular endothelial growth factor (VEGF, encoded by *VEGFA*) has been shown to be increased by mechanical stimulation, with this effect being thought to underlie the promotion of angiogenesis by such stimulation (Groothuis et al., 2010; Kasper et al., 2007; Weinbaum et al., 2011). Such upregulation of VEGF by mechanical stimulation has been detected in several cell types, including RPE cells (Farjood et al., 2020; Yehoshua et al., 2011). Structural changes associated with drusen formation are thought to give rise to mechanical stimulation of RPE cells. Such stimulation has the potential to induce excessive angiogenesis dependent on VEGF production by RPE cells (Farjood et al., 2020; Yehoshua et al., 2011). Several molecular mechanisms have been implicated in the regulation of VEGF expression in various cell types. In vascular endothelial cells, the transcriptional coactivator YAP (encoded by *YAP1*), ion channels of the transient receptor potential (TRP) family and the transcription factor hypoxia-inducible factor 1α (HIF-1α, encoded by *HIF1A*) have all been found to contribute to upregulation of VEGF expression (Ikeda et al., 1995; Smani et al., 2018; Wang et al., 2017). However, the molecular mechanisms by which mechanical stimulation elicits VEGF production in RPE cells have remained unclear. In this study, we examined the effects of mechanical stimulation on the expression of various angiogenic factors in RPE cells both *in vitro* and *in vivo* as well as investigated the mechanisms of such effects.

## RESULTS

### Induction of angiogenesis-related factors in RPE1 cells by mechanical stimulation

**Fig. 1.**
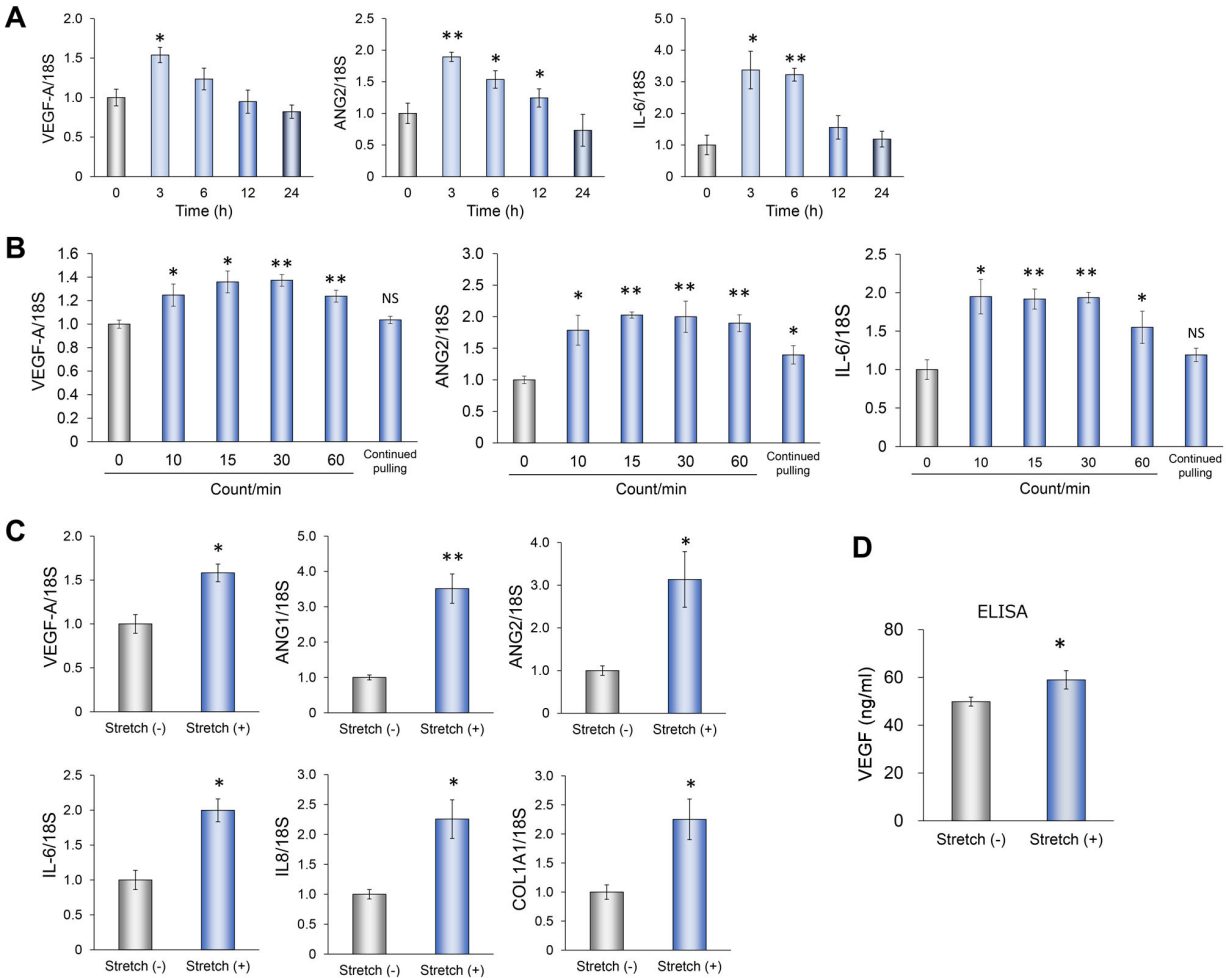
**Mechanical stimulation-induced expression of angiogenesis-related factor genes in RPE1 cells.** (A) qPCR analysis of *VEGFA*, *ANG2* and *IL6* mRNA abundance in RPE1 cells subjected to mechanical stimulation at 30 counts/min for the indicated times. Data were normalized to the amount of 18S rRNA and expressed relative to the corresponding value for non-stimulated cells. (B) qPCR analysis of the amounts of *VEGFA*, *ANG2* and *IL6* mRNAs in RPE1 cells subjected to mechanical stimulation at the indicated frequencies for 3 h. (C) qPCR analysis of *VEGFA*, *ANG2*, *IL6*, *ANG1*, *IL8* and *COL1A1* mRNA levels in RPE1 cells subjected (or not) to mechanical stimulation at 30 counts/min for 3 h. (D) ELISA analysis of the concentration of VEGF in culture supernatants of RPE1 cells subjected (or not) to mechanical stimulation at 30 counts/min for 3 h. All data are mean±s.e.m. for four independent experiments. NS, not significant; *P<0.05; **P<0.01; versus non-stretched control (Student's two-tailed paired *t*-test).

Mechanical stimulation has previously been shown to increase the expression of angiogenic factors including VEGF in RPE cells (Farjood et al., 2020; Farjood and Vargis, 2018; Wu et al., 2017). To investigate further the effects of mechanical stimulation on the expression of angiogenesis-related factors in RPE1 cells, we first examined changes in gene expression induced by such stimulation in human RPE1 cells cultured in stretch-stimulation chambers. Real-time quantitative PCR (qPCR) analysis revealed that the amounts of *VEGFA*, angiopoietin 2 (*ANG2*, also known as *ANGPT2*), and interleukin-6 (*IL6*) mRNAs were significantly increased after mechanical stimulation for 3 h and subsequently returned to basal levels despite continued stimulation for up to 24 h (Fig. 1A). Examination of the effect of the frequency of mechanical stimulation for 3 h showed that stimulation at each frequency (10, 15, 30 or 60 counts/min) induced similar increases in the abundance of *VEGFA*, *ANG2* or *IL6* mRNAs, whereas continued pulling of the cells was less effective (Fig. 1B). Subsequent experiments were therefore performed with mechanical stimulation for 3 h at 30 counts/min. In addition to *VEGFA*, *ANG2* and *IL6*, expression of the genes encoding interleukin -8 (IL-8, also known as CXCL8), collagen type 1 α1 (COL1A1) and angiopoietin 1 (ANG1, also known as ANGPT1), all of which also contribute to angiogenesis, was also significantly increased by mechanical stimulation of RPE1 cells (Fig. 1C). An enzyme-linked immunosorbent assay (ELISA) revealed that the effect of mechanical stimulation on VEGF expression was also apparent at the protein level (Fig. 1D).

### Role of HIF-1α in the effects of mechanical stimulation on angiogenesis-related gene expression in RPE1 cells

**Fig. 2.**
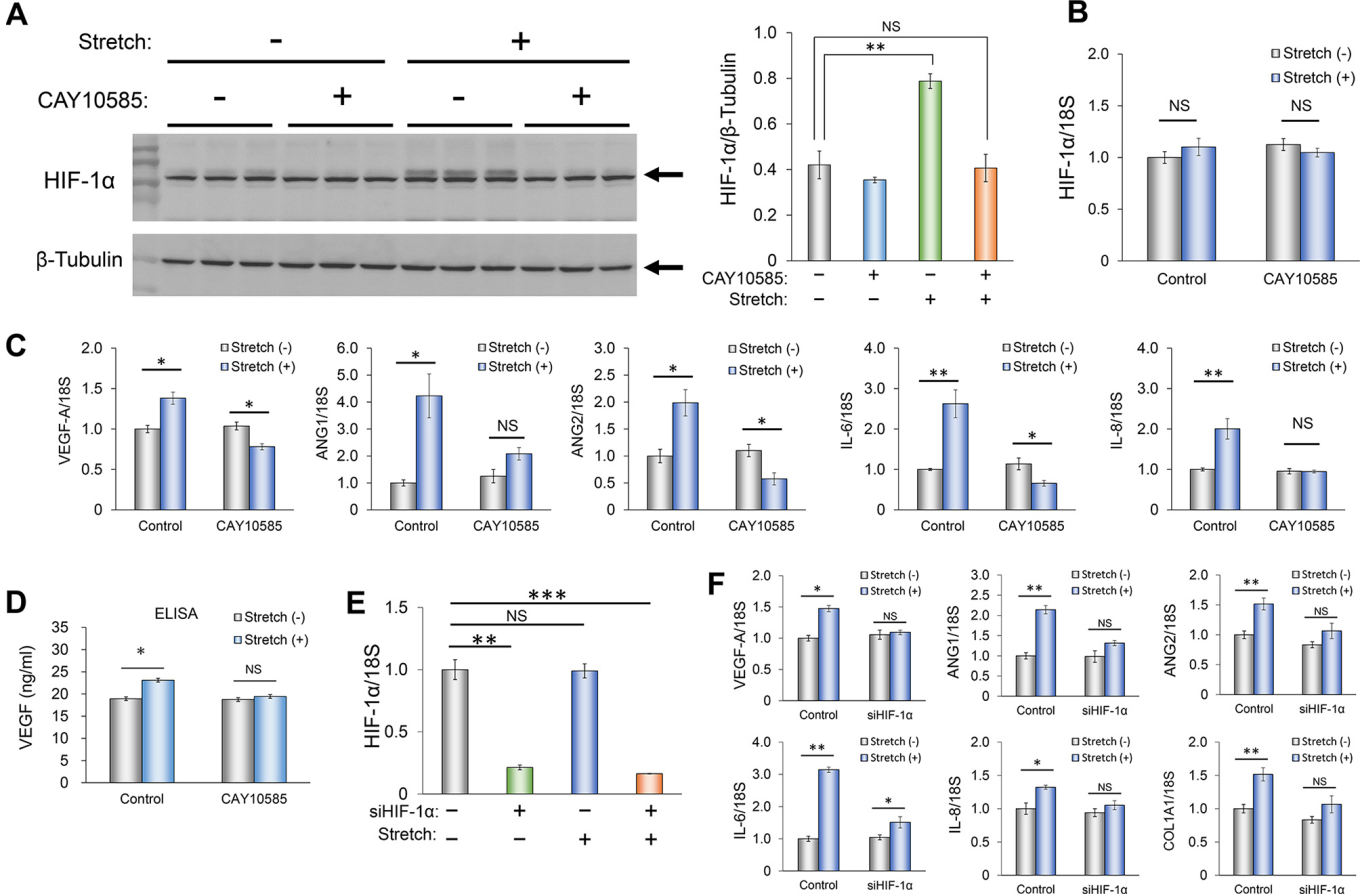
**Role of HIF-1α in the upregulation of angiogenesis-related gene expression by mechanical stimulation in RPE1 cells.** (A) Immunoblot analysis of HIF-1α in RPE1 cells subjected (or not) to mechanical stimulation for 3 h in the absence or presence of the HIF-1α inhibitor CAY10585 (left panel). Band intensity for HIF-1α in the three replicates shown was normalized to that for β-tubulin and presented as the mean±s.e.m. (right panel). (B,C) qPCR analysis of *HIF1A* mRNA (B) and *VEGFA*, *ANG1*, *ANG2*, *IL6* and *IL8* mRNAs (C) for cells treated as in A. Data were normalized to the amount of 18S rRNA and expressed relative to the corresponding value for non-stimulated cells not exposed to the inhibitor. (D) ELISA analysis of VEGF concentration in culture supernatants of cells treated as in A. (E,F) qPCR analysis of *HIF1A* mRNA (E) and *VEGFA*, *ANG1*, *ANG2*, *IL6*, *IL8* and *COL1A1* mRNAs (F) in RPE1 cells transfected with HIF-1α or control siRNAs and then subjected (or not) to mechanical stimulation for 3 h. Quantitative data are mean±s.e.m. NS, not significant; *P<0.05; **P<0.01; ***P<0.001 (Student's two-tailed paired *t*-test).

HIF-1α was identified as a transcription factor that binds specifically to an enhancer element of the erythropoietin (*EPO*) gene (Semenza and Wang, 1992; Wang and Semenzas, 1993) and was subsequently found to regulate the expression of various genes, including *VEGFA* (Ema et al., 1997; Semenza, 1998). It has also been implicated in the responses of cells to mechanical stimulation (Kakinuma et al., 2005; Kim et al., 2002; Milkiewicz et al., 2007). To test whether HIF-1α contributes to such responses in RPE1 cells, we examined the effects of the HIF-1α inhibitor CAY10585 (Lee et al., 2007, 2010). Immunoblot analysis revealed that mechanical stimulation increased the abundance of HIF-1α in RPE1 cells in a manner sensitive to inhibition by CAY10585 (Fig. 2A), whereas qPCR analysis showed that such stimulation had no effect on the amount of *HIF1A* mRNA (Fig. 2B). These results suggested that the HIF-1α protein is stabilized by mechanical stimulation, consistent with previous findings (Kim et al., 2002). qPCR analysis also revealed that the mechanical stimulation-induced upregulation of *VEGFA*, *ANG1*, *ANG2*, *IL6* and *IL8* mRNAs was abolished by treatment with CAY10585 (Fig. 2C), as was the associated increase in the amount of VEGF protein in culture supernatants (Fig. 2D). Furthermore, siRNA-mediated knockdown of HIF-1α in RPE1 cells (Fig. 2E) also greatly attenuated the effects of mechanical stimulation on the expression of angiogenesis-related factor genes (Fig. 2F). Collectively, these results indicated that HIF-1α plays a key role in the upregulation of angiogenesis-related factors by mechanical stimulation in RPE1 cells.

### Roles of SRC and p38 MAPK in the upregulation of angiogenic factors by mechanical stimulation in RPE1 cells

**Fig. 3.**
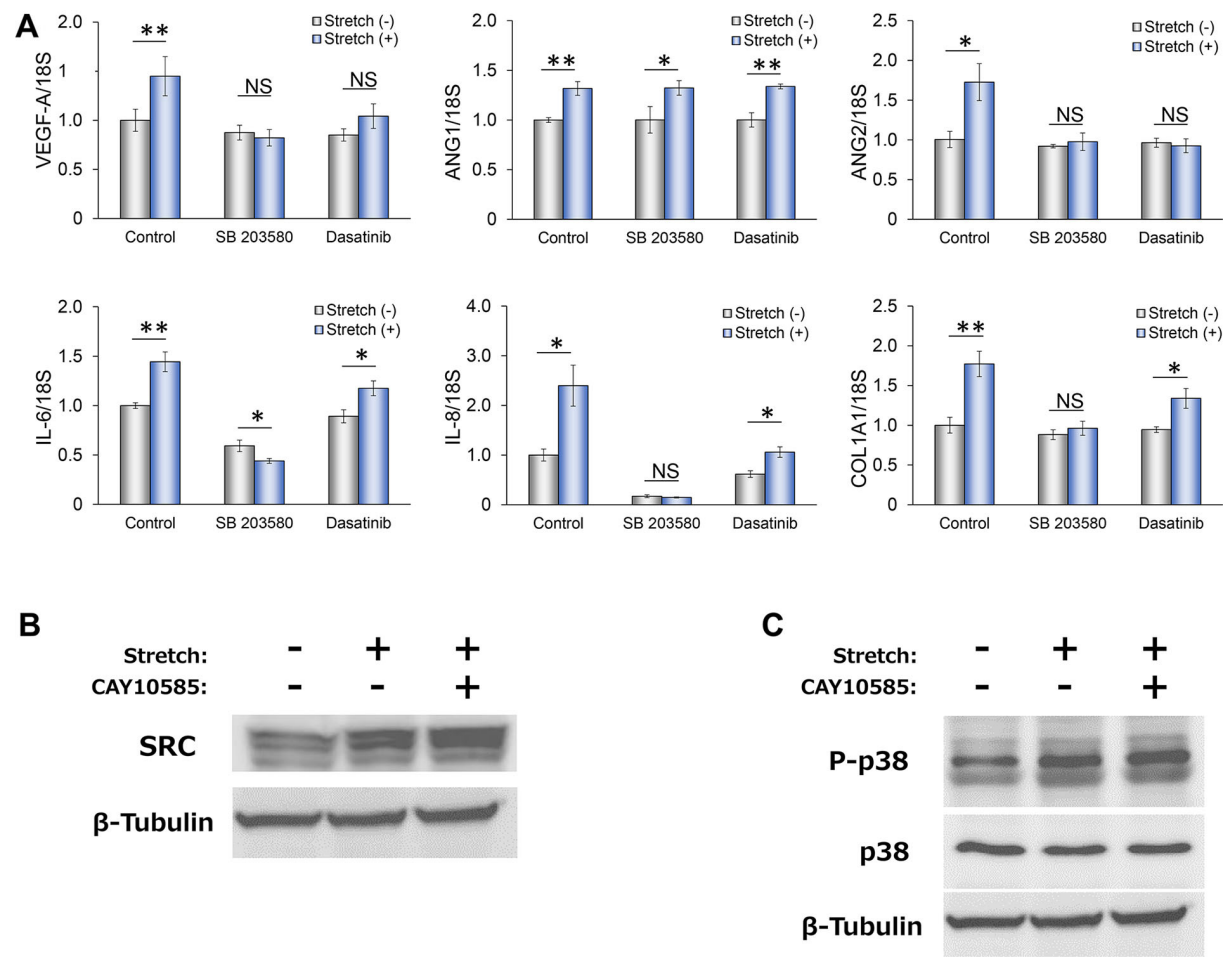
**Roles of SRC and p38 MAPK in the HIF-1α-dependent upregulation of angiogenesis-related gene expression by mechanical stimulation in RPE1 cells.** (A) qPCR analysis of *VEGFA*, *ANG1*, *ANG2*, *IL6* and *IL8* mRNA levels in RPE1 cells subjected (or not) to mechanical stimulation for 3 h in the absence or presence of the p38 inhibitor SB203580 or the SRC inhibitor dasatinib. Data were normalized to the amount of 18S rRNA and expressed relative to the corresponding value for non-stimulated cells not exposed to inhibitor. Data are means±s.e.m. for four independent experiments. NS, not significant; *P<0.05; **P<0.01 (Student's two-tailed paired *t*-test). (B,C) Immunoblot analysis of SRC (B) and phosphorylated (P-) and total p38 MAPK (C) in RPE1 cells subjected (or not) to mechanical stimulation for 3 h in the absence or presence of the HIF-1α inhibitor CAY10585. Data are representative of three independent experiments.

Several signaling pathways have been implicated in the HIF-1α-mediated upregulation of gene expression, including expression of *VEGFA* (Ema et al., 1997; Semenza, 1998). We therefore examined the potential roles of various such pathways in our experimental system. We first investigated the potential contribution of the transcriptional coactivators YAP and TAZ (also known as tafazzin) (Dupont et al., 2011; Wada et al., 2011), which function downstream of the Hippo signaling pathway and have been implicated in mechanotransduction. Although mechanical stimulation induced activation of YAP in RPE1 cells (Fig. S1A), the upregulation of angiogenesis-related gene expression by such stimulation was not affected by knockdown of YAP, TAZ or both proteins (Fig. S1B,C). The increases in the abundance of *VEGFA*, *ANG2* and *IL6* mRNAs induced by mechanical stimulation in RPE1 cells were also unaffected by inhibitors of the mechanosensitive channel Piezo1 (Coste et al., 2010; Suchyna, 2017) or of TRP channels (Clapham, 2003; Montell and Rubin, 1989; Wong et al., 1989) (Figs S2 and S3). The tyrosine kinase SRC has been shown to activate the p38 mitogen-activated protein kinase (MAPK) pathway in response to mechanical stimulation (Han et al., 2004; Liu et al., 1996; Sawada et al., 2001; Tamada et al., 2004). We therefore tested whether SRC or p38 MAPK might contribute to the mechanical stimulation-induced expression of angiogenic factors in RPE1 cells. The SRC inhibitor dasatanib (O'hare et al., 2005; Shah et al., 2006) and the p38 inhibitor SB203580 each abolished or greatly attenuated the upregulation of angiogenic factor gene expression induced by mechanical stimulation in RPE1 cells (Fig. 3A). In contrast, as dasatanib has been reported to inhibit a wide range of phosphorylation (Rix et al., 2007), we used another SRC inhibitor, bosutinib, which, similar to dasatanib, inhibited increases in *VEGFA* and *ANG2* expression after mechanical stimulation (Fig. S4). Furthermore, mechanical stimulation increased the abundance of SRC (Fig. 3B) and induced the phosphorylation (activation) of p38 MAPK (Fig. 3C) in RPE1 cells, and these effects were not influenced by the HIF-1α inhibitor CAY10585.

### Induction of angiogenesis by factors released from RPE1 cells in response to mechanical stimulation

**Fig. 4.**
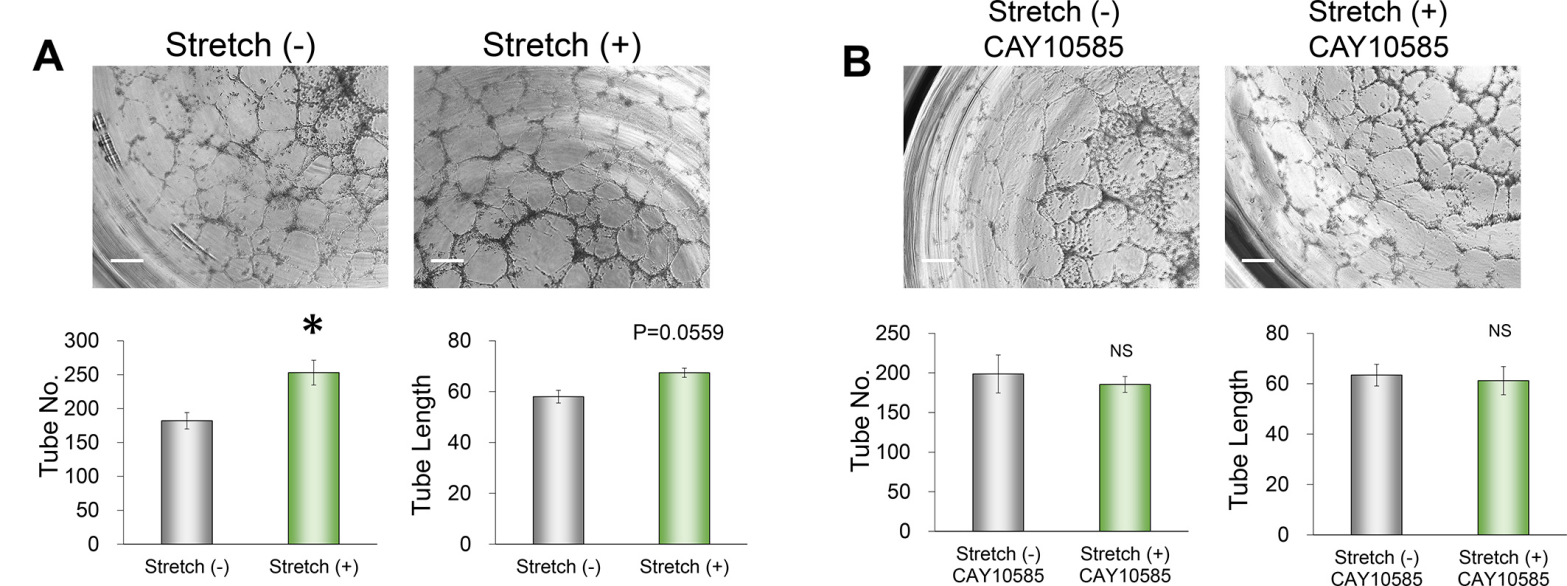
**Angiogenesis induced by factors released from RPE1 cells in response to mechanical stimulation.** (A,B) Light microscopy images of tube formation by vascular endothelial cells (top) as well as quantitation of tube length and number (bottom). Assays were performed in the presence of culture supernatants of RPE1 cells subjected (or not) to mechanical stimulation for 3 h, and without the inhibitor (A) or with CAY10585 (B). Scale bars: 300 µm. The quantitative data are means±s.e.m. for five independent experiments. NS, not significant; *P<0.05 (Student's two-tailed paired *t*-test).

To investigate the effect of mechanical stimulation of the retinal pigment epithelium on angiogenesis, we examined the effect of culture supernatants of RPE1 cells subjected to mechanical stimulation on tube formation by vascular endothelial cells. Such culture supernatants increased the number of tubes formed by the endothelial cells compared with that apparent in the presence of culture supernatants from non-stimulated RPE1 cells (Fig. 4A). Tube length also tended to be increased by exposure of the endothelial cells to the culture supernatants from mechanically stimulated RPE1 cells (Fig. 4A). Furthermore, treatment of RPE1 cells with the HIF-1α inhibitor CAY10585 before mechanical stimulation greatly attenuated the effect of the culture supernatants on tube formation (Fig. 4B). These results thus indicated that mechanical stimulation induces the release of functional angiogenic factors from RPE1 cells in a manner dependent on HIF-1α.

### HIF-1α-dependent induction of angiogenesis-related gene expression by mechanical stimulation *in vivo*

**Fig. 5.**
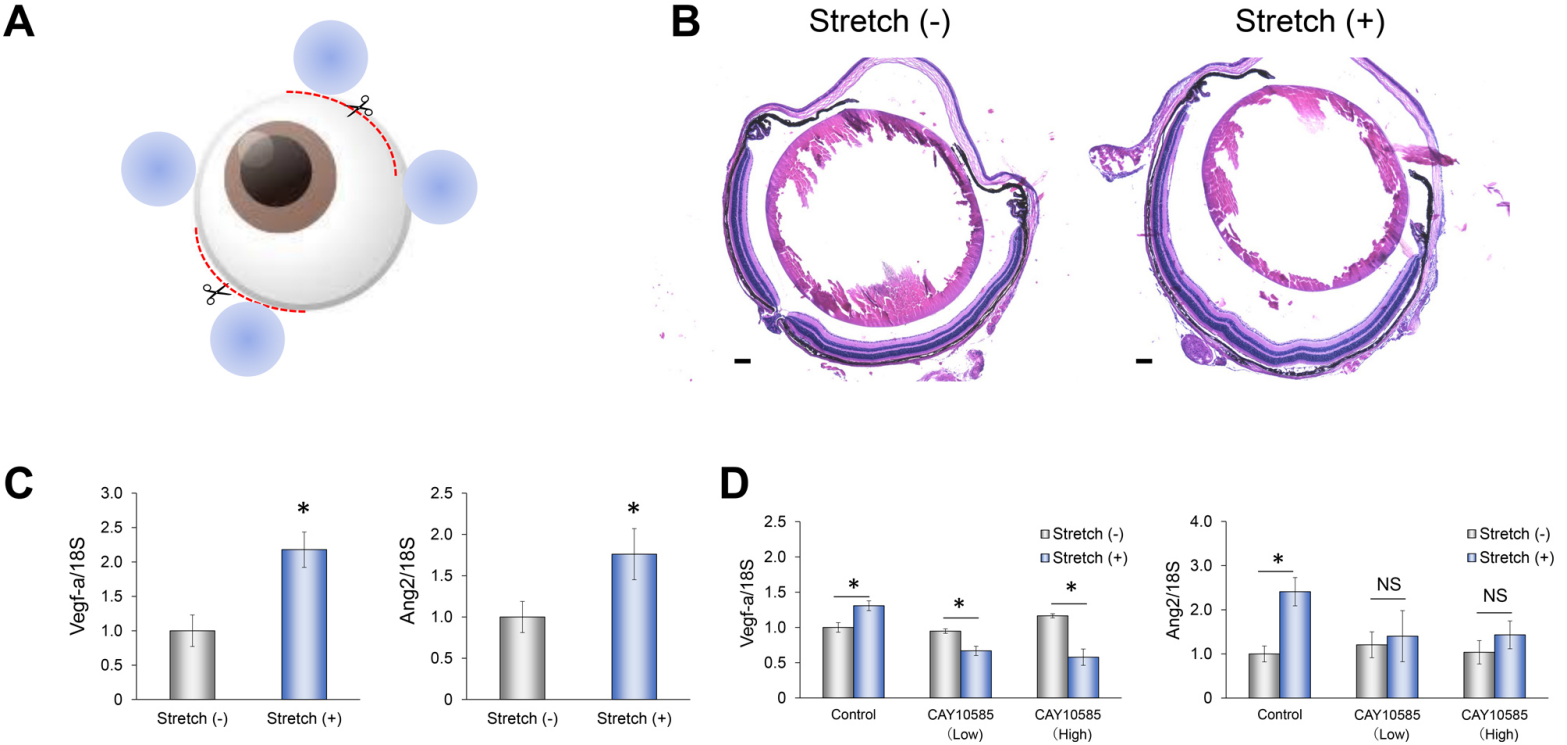
**Role of HIF-1α in regulation of angiogenesis-related gene expression in RPE cells by mechanical stimulation in a mouse model.** (A) *In vivo* physical stimulation model. Two incisions were made in the conjunctiva and two beads were inserted into each incision and sutured in place. (B) Hematoxylin and Eosin staining of a control eye (left) and an eye at 2 days after bead insertion (right). Images are representative of ten eyes. Scale bars: 100 µm. (C) qPCR analysis of *Vegfa* and *Ang2* mRNA levels in RPE cells isolated from control eyes or eyes at 2 days after bead insertion. (D) qPCR analysis of *Vegfa* and *Ang2* mRNA levels in RPE cells isolated from control eyes or mechanically stimulated eyes that had been injected with the HIF-1α inhibitor CAY10585 at doses of 1 µg (low) or 10 µg (high) at the same time as bead insertion. All quantitative data were normalized to the amount of 18S rRNA and expressed relative to the corresponding control value, and are mean±s.e.m. for five eyes. NS, not significant; *P<0.05; **P<0.01 (Student's two-tailed paired *t*-test).

Given that our results revealed the importance of HIF-1α in the regulation of angiogenic factor expression by mechanical stimulation in RPE1 cells *in vitro*, we attempted to validate this role of HIF-1α with a mouse model *in vivo*. To mimic the effects of drusen formation, we developed a mechanical stimulation model based on insertion of glass beads into conjunctival incisions for 2 days (Fig. 5A). Hematoxylin and Eosin (H&E) staining revealed no abnormal histological changes in eyes subjected to this procedure (Fig. 5B). RPE cells were isolated from the manipulated and control eyes for analysis of gene expression. RPE cells from eyes subjected to mechanical stimulation showed significant increases in the amounts of *Vegfa* and *Ang2* mRNAs (Fig. 5C). Furthermore, intraocular injection of the HIF-1α inhibitor CAY10585 prevented these effects of mechanical stimulation on *Vegfa* and *Ang2* gene expression (Fig. 5D). These results thus implicated HIF-1α in upregulation of angiogenesis-related gene expression by mechanical stimulation in RPE cells *in vivo* as well as *in vitro*, showing that such upregulation can be attenuated by pharmacological inhibition of HIF-1α.

## DISCUSSION

Here, we characterized the effects of mechanical stimulation on the expression of angiogenic factors in RPE cells at the molecular level. Mechanical stimulation of cultured RPE1 cells increased the expression of angiogenesis-related factors such as VEGF in a manner dependent on HIF-1α. The activation of HIF-1α by mechanical stimulation in turn appeared to be mediated by SRC and p38 MAPK signaling, with inhibitors of these signaling molecules found to attenuate the upregulation of angiogenesis-related gene expression by mechanical stimulation. Finally, we showed that physical stimulation increased the expression of the *Vegfa* and *Ang2* genes in RPE cells in a mouse model, and that this action was blocked by intraocular injection of a pharmacological inhibitor of HIF-1α.

The effects of mechanical stimulation on RPE cells in culture have been studied previously. Elongation stimulation from the cell base in a dedicated chamber induced a local increase in the extracellular concentration of VEGF, and stimulation in a stretch chamber also increased VEGF expression (Farjood and Vargis, 2018; Yehoshua et al., 2011). Such studies have attempted to reproduce *in vitro* the effects of the physical compression of the RPE cell layer from the base induced by drusen. The finding that physical stimulation upregulates VEGF expression in RPE cells has led to the proposal that this effect gives rise to the exaggerated angiogenesis that underlies the development of diseases such as AMD. However, none of these previous studies has characterized the detailed molecular mechanisms underlying the effects of mechanical stimulation on angiogenic factor expression in RPE cells *in vitro*, and the relevance of culture models to pathological angiogenesis *in vivo* has remained uncertain. Our results have now addressed both of these issues.

HIF-1α is a transcription factor that regulates the expression of various genes, including *VEGFA*, in response to signals such as hypoxia. YAP, one of the typical mechanical stimulation sensors, has been reported to interact with HIF-1α and was predicted to be involved in the increase in expression of *VEGFA* and other genes following mechanical stimulation of RPE1 cells (Zhang et al., 2018). However, although we found that mechanical stimulation activated YAP in RPE1 cells, knockdown of YAP or of the related factor TAZ did not affect the upregulation of angiogenesis-related gene expression induced by such stimulation. Given that the response to mechanical stimulation varies with cell type and scaffold environment, such differences may account for apparent discrepancies in the contribution of YAP to the effects of such stimulation. The SRC–p38 MAPK pathway has also been implicated as a transducer of the effects of mechanical stimulation and in the activation of HIF-1α (Sawada et al., 2001). Indeed, we found that mechanical stimulation increased SRC expression and p38 phosphorylation in RPE1 cells, and that inhibitors of these kinases attenuated both HIF-1α activation and angiogenic factor expression induced by such stimulation. Our results are thus consistent with the operation of a SRC–p38–HIF-1α axis in the induction of angiogenesis-related gene expression in RPE1 cells by mechanical stimulation.

We found that inhibition of HIF1-α function, either by CAY10585 treatment or siRNA transfection, did not attenuate the expression of *VEGFA* or other genes in RPE1 cells under basal conditions, but only did so in mechanically stimulated cells. These results suggest that HIF-1α does not contribute to the regulation of angiogenic factor expression at the steady state but is required specifically for the upregulation of such factor expression by mechanical stimulation. Expression of VEGF and other angiogenic factors at the steady state is important for the maintenance of retinal structure, with suppression of such expression likely to be detrimental to retinal homeostasis. This selective contribution of HIF-1α to angiogenesis induced by mechanical stimulation is potentially beneficial from the clinical viewpoint of the application of pharmacological inhibitors of HIF-1α to the treatment of pathological angiogenesis.

RPE cells phagocytose photoreceptor cells to maintain visual homeostasis. The process of phagocytosis involves active morphological changes by the cells themselves, such as contraction of F-actin, which may induce mechanical stimulation of the cells. In addition, phagocytosis has distinct diurnal variations and is highest in the early morning (Lakkaraju et al., 2020; LaVail, 1976); in the *in vivo* experiments, the operation was performed during a fixed time in the evening to suppress the effect of phagocytosis and reduce the variation between experiments. In contrast, lipofuscin accumulated during aging forms drusen, which accumulates in the basal layer of the retinal pigment epithelium. Accumulation of drusen leads to local retinal pigment epithelium compression and scaffold instability of the basal layer. These external environmental changes provide passive mechanical stimuli to the retinal pigment epithelium, which in turn provides the retinal pigment epithelium with a condition that facilitates constant stimulation and may contribute to the promotion of angiogenesis. In contrast, it has been reported that active mechanical stimulation and passive mechanical stimulation activate different signaling pathways (Polyakov et al., 2014; Ricca et al., 2013). This study focused on passive mechanical stimuli, but the effects of active mechanical stimuli such as phagocytosis remain unclear. Active mechanical stimulation is difficult to verify experimentally, and this is an issue that needs to be resolved in the future.

Mechanical stimulation of RPE cells is assumed to affect the physical environmental changes associated with drusen formation. However, there are AMDs that do not form drusen, and the pathological effects of the presence or absence of drusen have not been fully elucidated. The focus of the present study on mechanical stimulation is expected to improve our understanding of the pathophysiology of drusen-type AMD. Mimicking non-drusen type AMD is technically difficult, and this research is a future challenge. The results of this study will be an important insight in understanding the differences in the pathophysiology of the drusen and non-drusen types of AMD.

The retinal pigment epithelium *in vivo* forms a strong tight junction. This essentially acts as a barrier to prevent choroidal vessels from entering the retinal side. Previous reports have shown that ANG2 can cause the tight junctions to diverge (Rangasamy et al., 2011). Therefore, mechanical stimulation *in vivo* may not only induce direct angiogenesis, but may also have indirect angiogenic effects due to the disruption of retinal pigment epithelium barrier function. In contrast, RPE1 cells do not express ZO-1 (also known as TJP1) and other tight junction components and do not form an epithelial barrier. This suggests that mechanical stimulation can induce angiogenesis regardless of the presence or absence of a barrier. However, the effect of stretching on barrier performance remains unclear; the influence of mechanical stimulation on AMD is an area that should be studied from a more complex perspective. The direct regulation of angiogenic factors by mechanical stimulation in this study may help to understand these mechanisms.

Mechanical stimulation increased the expression of the *IL6*, *IL8* and *ANG2* genes as well as that of the *VEGFA* gene. These factors not only induce angiogenesis but may trigger the epithelial–mesenchymal transition, which has been implicated in disruption of the RPE layer. It is therefore possible that changes to the physical environment of RPE cells as a result of drusen formation not only induce angiogenesis, but also disrupt the barrier function of RPE cells, thereby generating an environment more conducive to the invasion of blood vessels. The upregulation of these factors by mechanical stimulation revealed in this study may thus provide a basis for further characterization of the relation between AMD progression and the epithelial–mesenchymal transition.

## MATERIALS AND METHODS

### Cell culture

Human retinal pigment epithelial (RPE1) cells were cultured under 5% CO_2_ at 37°C in Dulbecco's modified Eagle medium: nutrient mixture F-12 (DMEM/F12; Gibco, 11320033) supplemented with 10% fetal bovine serum (Gibco, 10270-106) and 1% antibiotic-antimycotic (Gibco, 15240062). RPE1 is an immortalized human RPE cell line stably transfected with a vector for the human telomerase reverse transcriptase. The cells are distributed by American Type Culture Collection (Manassas, VA, USA).

### Reagents

The HIF-1α inhibitor CAY10585 (ab144422) was obtained from Abcam, the p38 MAPK inhibitor adezmapimod (SB203580) from MedChemExpress, the SRC inhibitors dasatinib (S1021) and bosutinib (S1014) from Selleck, the Piezo1 inhibitor GsMTx4 (4393-s) from Peptide Institute, and the TRP channel inhibitors gadolinium chloride hexahydrate (078-02661) and ruthenium red (185-03183) from Fujifilm Wako. CAY10585, SB203580, dasatinib and ruthenium red were added to the medium at 10 µM, GsMTx4 at 5 µM, and gadolinium chloride hexahydrate at 100 µM.

### RNA interference

Small interfering RNAs (siRNAs) specific for HIF-1α (HSS104774), YAP (HSS115944) or TAZ (HSS119545) as well as a control siRNA (45-2001) were obtained from Invitrogen. Cells were transfected with the siRNAs at 100 µM with the use of the Lipofectamine RNAiMAX reagent (Invitrogen).

### Mechanical stimulation of cells *in vitro*

Stretch chambers (Strex, STB-CH-04) coated with 0.5 ml/well of 10% native collagen acidic solution (Koken, I-AC-50) were kept at room temperature for 1 day and excess collagen was washed out with PBS. 1×10^5^ RPE1 cells/well were seeded into stretch chambers and cultured for 1 day. RPE1 cells were subjected to mechanical stimulation with the use of an automated cell stretching system (Strex, STB-1400) placed in an incubator at 37°C containing 5% CO_2_. Unless indicated otherwise, stimulation was performed at a frequency of 30 counts/min with an extension distance of 10% and duration of 3 h. RPE1 cells subjected to mechanical stimulation were observed under a microscope, confirming that adhesion to the stretch chamber was maintained.

### qPCR analysis

Total RNA was extracted from cells with the use of Sepasol RNA I Super G (Nacalai Tesque, 09379-55). The isolated RNA (500 ng) was subjected to reverse transcription with the use of ReverTra Ace qPCR RT Master Mix with gDNA Remover (Toyobo, FSQ-301), and the resulting cDNA was subjected to qPCR analysis with PowerUp SYBR Green Master Mix (Applied Biosystems, A25742) and a StepOne real-time PCR system (Applied Biosystems). The sequences of the primers for qPCR are provided in Table S1. The abundance of each target mRNA was calculated by the comparative CT method and normalized by that of 18S rRNA.

### Immunoblot analysis

RPE1 cells were lysed for 20 min on ice in radioimmunoprecipitation (RIPA) buffer (20 mM Tris-HCl at pH 7.4, 1 mM EDTA, 150 mM NaCl, 1% Nonidet P-40, 0.1% SDS) supplemented with a protease inhibitor cocktail (Nacalai Tesque), and the lysates were centrifuged at 12,000 ***g*** for 10 min at 4°C. The resulting supernatants were assayed for protein concentration with a Pierce BCA Protein Assay Kit (Thermo Fisher Scientific, 23227) before fractionation by SDS-PAGE on a 5-20% e-PAGEL gel (Atto) at 20 mA for 80 min. The separated proteins were transferred to a 0.45-µm cellulose nitrate membrane (Cytiva), which was then exposed to 2% bovine serum albumin (BSA; Sigma-Aldrich) or 5% skim milk (Fujifilm Wako) at room temperature before incubation overnight at 4°C with antibodies against YAP (Abcam, ab52771, 1:1000), TAZ (Abcam, ab119373, 1:1000), HIF-1α (Abcam, ab51608, 1:500), SRC (Cell Signaling Technology, 2108, 1:1000), p38 (Cell Signaling Technology, 9212, 1:1000), phospho-p38 (Cell Signaling Technology, 9211, 1:500) or β-tubulin (Cell Signaling Technology, 2128, 1:5000) in the same blocking solution. The membrane was washed with TBS containing 0.05% Tween 20 (Fujifilm Wako) and then incubated for 1 h at room temperature with anti-mouse IgG, HRP-linked F (GE Healthcare, NA9310V) or anti-rabbit IgG, HRP-linked F (GE Healthcare, NA9340V) immunoglobulin G diluted in blocking solution. Immune complexes were detected with the use of Amersham ECL Prime (Cytiva) or Chemi-Lum One Super (Nacalai Tesque) and with a ChemiDoc Imaging System (Bio-Rad). Band intensity was quantified with the use of ImageJ software.

### Immunostaining

After mechanical stimulation, cells were washed with PBS, fixed with 4% paraformaldehyde (Muto Pure Chemicals) for 15 min at room temperature, and exposed first to 0.5% Tween 20 for 10 min and then to 1% BSA for 1 h at room temperature, before incubation overnight at 4°C with antibodies against active YAP (Abcam, ab205270) diluted 1:500 in 1% BSA. The cells were washed five times with 0.1% Tween 20 in PBS, incubated for 1 h at room temperature with Alexa Fluor 488-conjugated secondary antibodies to rabbit immunoglobulin G (Invitrogen, A-11008) diluted 1:5000 in 1% BSA, washed again, and observed with an all-in-one fluorescence microscope (Keyence, BZ-X710). The nuclear/cytoplasmic fluorescence ratio for active YAP was determined with ImageJ software.

### ELISA

Culture supernatants from mechanically stimulated RPE1 cells were centrifuged at 300 ***g*** for 5 min to remove debris before assaying for VEGF with the Human VEGF Quantikine ELISA Kit (R&D Systems).

### Mechanical stimulation of RPE cells *in vivo*

C57BL/6J mice at 8 weeks of age were obtained from Japan SLC (Shizuoka, Japan). Mice were anesthetized with combined triadic anesthesia. Each drug was diluted in clean PBS, and 0.75 mg/kg medetomidine hydrochloride (Domitor; Nippon zenyaku kogyo), 4 mg/kg midazolam (Dormicum, Maruishi Pharmaceutical) and 5 mg/kg butorphanol tartrate (Betlfal, Meiji Animal Health) were administered intraperitoneally. The upper and lower eyelids of one eye of the mice were incised without damaging the external ocular muscles. Two clean glass beads with a diameter of 0.5 mm were inserted into the incised conjunctiva of each eyelid and were secured by ligation with 10-0 nylon thread. 2 µl of CAY10585 or PBS was carefully administered intravitreally to avoid damage to the lens or retina. The eye was removed 48 h after bead insertion, and the retinal pigment epithelium was dissected for isolation of RPE cells as previously described (Kobayashi et al., 2021). These operations were performed between 17:00 and 20:00 with the aim of eliminating the effects of phagocytosis and other diurnal variations. The mouse experiments were approved by the Animal Ethics Committee of Yamaguchi University Graduate School of Medicine and were performed in accordance with the relevant guidelines and regulations. This study complies with the ARRIVE guidelines for animal research as well as with the ARVO Statement for the Use of Animals in Ophthalmic and Vision Research.

### H&E staining

The eyes of the excised mice were fixed by TB-Fix [3.7% formaldehyde (Wako, 064-00406), 15% ethanol (Wako, 057-00456), 10% acetic acid (Wako, 017-00256)], as previously reported (Tokuda et al., 2018). Briefly, the eyes were kept overnight at 4°C in PBS containing 10% formaldehyde, 10% ethanol and 10% acetic acid. Fixed eyes were embedded with paraffin and sectioned to a thickness of 5 µm using a SM 200R microtome (Leica). Slices dried on glass slides were stained with H&E (Merck Millipore) after the paraffin was removed and dehydrated with alcohol and xylene. These sections were mounted using Marinol (Muto Pure Chemicals).

### Statistical analysis

Data are presented as mean±s.e.m. and were analyzed by Student's two-tailed paired *t*-test. A *P*-value of <0.05 was considered statistically significant.

## Supplementary Material

10.1242/dmm.050640_sup1 Supplementary information
