# Supplementary material for: HIF-1α-dependent upregulation of angiogenic factors by mechanical stimulation in retinal pigment epithelial cells
Source: Dis Model Mech. 2024 May 1;17(4):dmm050640. doi: 10.1242/dmm.050640 (PMC11095633; doi:10.1242/dmm.050640)
Supplement: Supplementary information [file dmm-17-050640-s1.pdf]

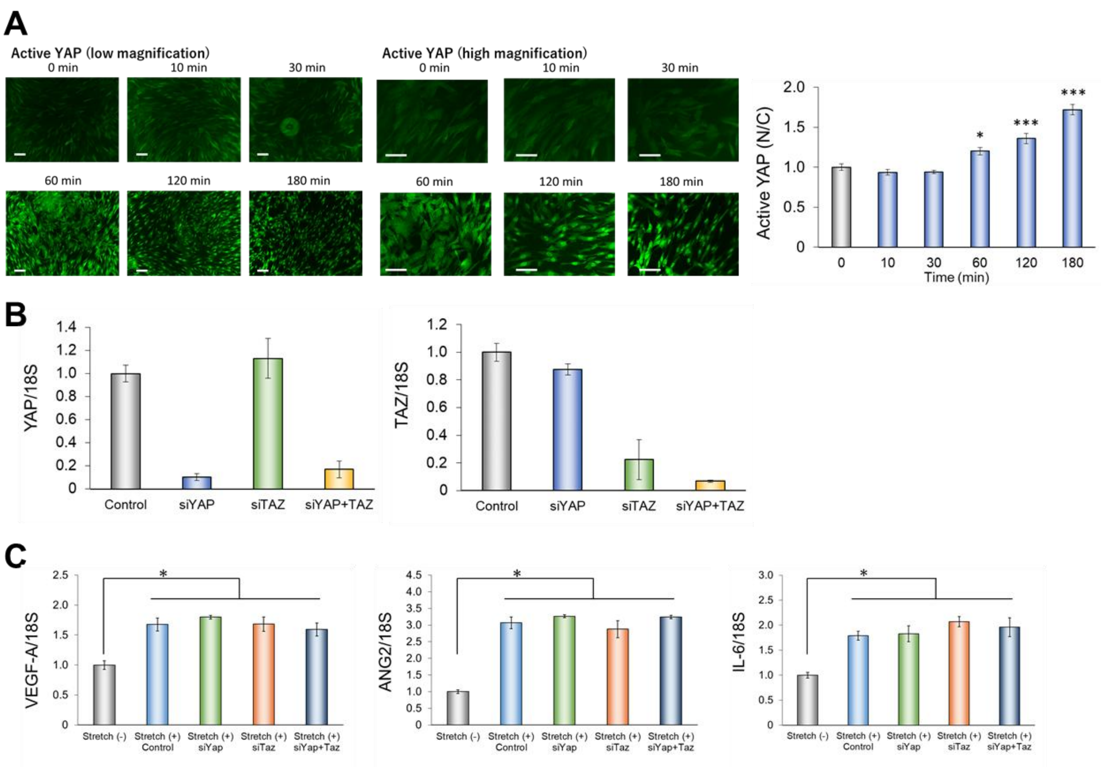


**Fig. S1. YAP and TAZ are not required for the upregulation of angiogenic factor gene expression by mechanical stimulation in RPE1 cells.** (**A**) Immunofluorescence staining of active YAP in RPE1 cells subjected to mechanical stimulation for the indicated times. Representative images at low and high magnification as well as the relative nuclear/cytoplasmic (N/C) ratio for active YAP determined as mean ± SEM values from four independent experiments are shown. Scale bars, 100 and 50 μm for low and high magnification, respectively. (**B**) Knockdown efficiency for YAP and TAZ in RPE1 cells evaluated by qPCR analysis. Data were normalized by the amount of 18S rRNA and expressed relative to the corresponding value for cells transfected with the control siRNA (**C**) qPCR analysis of VEGF-A, ANG2, and IL-6 mRNA levels in RPE1 cells transfected with control, YAP, or TAZ siRNAs, as indicated, and then subjected to mechanical stimulation for 3 h. Data were normalized by the amount of 18S rRNA and expressed relative to the corresponding value for nonstimulated and nontransfected cells. Data are means ± SEM for three (**B**) or four (**C**) independent experiments. *$P < 0.05$, ***$P < 0.001$ (Student's two-tailed *t* test).

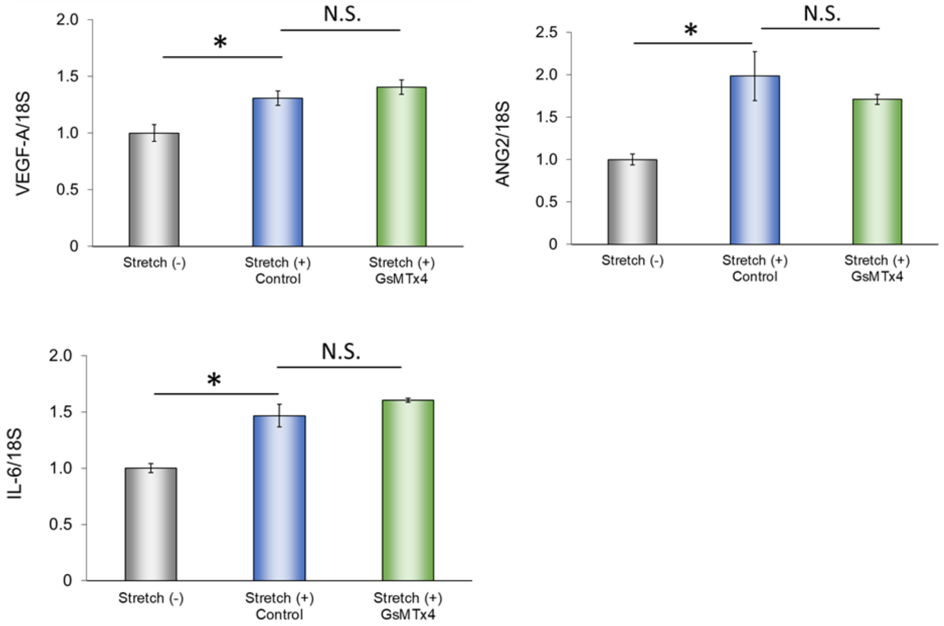


**Fig. S2**. **Lack of effect of a Piezo1 inhibitor on the upregulation of angiogenesis-related gene expression by mechanical stimulation in RPE1 cells.** RPE1 cells were subjected (or not) to mechanical stimulation for 3 h in the absence or presence of the Piezo1 inhibitor GsMTx4, after which the amounts of VEGF-A, ANG2, and IL-6 mRNAs were determined by qPCR analysis. Data were normalized by the amount of 18S rRNA and expressed relative to the corresponding value for nonstimulated cells. Data are means ± SEM for three independent experiments. *$P <$ 0.05, NS (Student's two-tailed $t$ test).

Disease Models & Mechanisms • Supplementary information

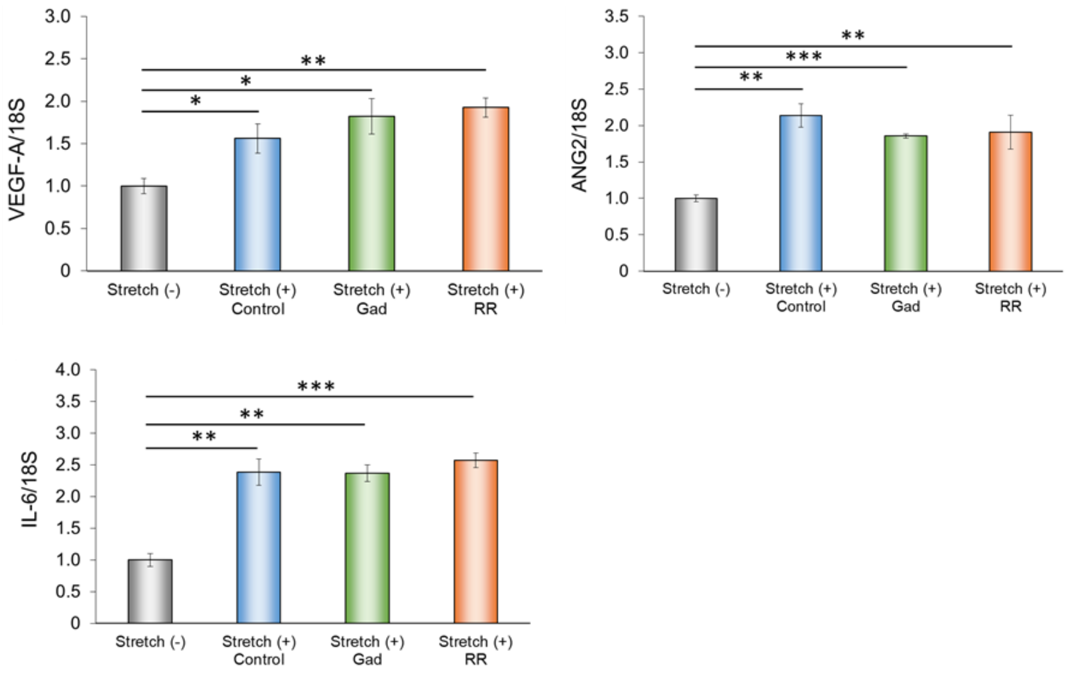


**Fig. S3. Lack of effect of TRP channel inhibitors on the upregulation of angiogenesis-related gene expression by mechanical stimulation in RPE1 cells.** RPE1 cells were subjected (or not) to mechanical stimulation for 3 h in the absence or presence of the TRP channel inhibitors gadolinium chloride hexahydrate (Gad) or ruthenium red (RR), after which the amounts of VEGF-A, ANG2, and IL-6 mRNAs were determined by qPCR analysis. Data were normalized by the amount of 18S rRNA and expressed relative to the corresponding value for nonstimulated cells. Data are means ± SEM for three independent experiments. *$P < 0.05$, **$P < 0.01$, ***$P <$ 0.001 (Student's two-tailed $t$ test).

Disease Models & Mechanisms • Supplementary information

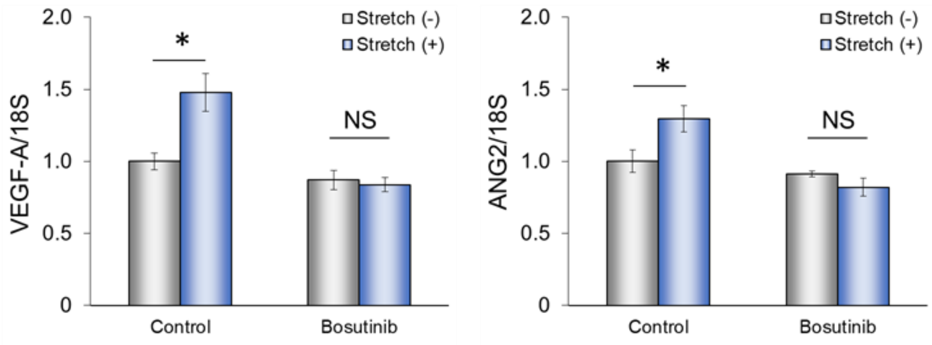


**Fig. S4. Suppression of angiogenic factors after mechanical stimulation with Bosutinib, an Src inhibitor.**

qPCR analysis of VEGF-A and ANG2 mRNA levels in RPE1 cells subjected (or not) to mechanical stimulation for 3 h in the absence or presence of the SRC inhibitor bosutinib. Data were normalized by the amount of 18S rRNA and expressed relative to the corresponding value for nonstimulated cells not exposed to inhibitor. Data are means ± SEM for four independent experiments. *$P < 0.05$, **$P < 0.01$, NS (Student's two-tailed *t* test).

Disease Models & Mechanisms • Supplementary information

**Table S1. Sequences of forward (F) and reverse (R) primers for qPCR analysis.**

| Gene | Sequence (5'→3') |
|---|---|
| VEGF-A (F) | GCAGAATCATCACGAAGTGGT |
| VEGF-A (R) | ACCAACGTACACGCTCCAG |
| IL-6 (F) | ACTCACCTCTTCAGAACGAATTG |
| IL-6 (R) | CCATCTTTGGAAGGTTCAGGTTG |
| IL-8 (F) | TTTTGCCAAGGAGTGCTAAAGA |
| IL-8 (R) | AACCCTCTGCACCCAGTTTTC |
| ANG1 (F) | AGCGCCGAAGTCCAGAAAAC |
| ANG1 (R) | TACTCTCACGACAGTTGCCAT |
| ANG2 (F) | AACTTTCGGAAGAGCATGGAC |
| ANG2 (R) | CGAGTCATCGTATTCGAGCGG |
| COL1A1 (F) | GAGGGCCAAGACGAAGACATC |
| COL1A1 (R) | CAGATCACGTCATCGCACAAC |
| YAP (F) | TAGCCCTGCGTAGCCAGTTA |
| YAP (R) | TCATGCTTAGTCCACTGTCTGT |
| TAZ (F) | GATCCTGCCGGAGTCTTTCTT |
| TAZ (R) | CACGTCGTAGGACTGCTGG |
| HIF-1α (F) | GAACGTCGAAAAGAAAAGTCTCG |
| HIF-1α (R) | CCTTATCAAGATGCGAACTCACA |
| 18S rRNA (F) | CGCCGCTAGAGGTGAAATTC |
| 18S rRNA (R) | CGAACCTCCGACTTTCGTTCT |

Disease Models & Mechanisms • Supplementary information
